# Pediatric primary splenic angiosarcoma: an aggressive multidisciplinary approach to the oncologic management of a rare malignancy

**DOI:** 10.1186/1477-7819-12-379

**Published:** 2014-12-09

**Authors:** Oscar K Serrano, Esther Knapp, Kevin Huang, Galina Baran, Mindy Statter, Danielle McClain, Jonathan Gill

**Affiliations:** Division of Pediatric Surgery, Department of Surgery, Children’s Hospital at Montefiore Medical Center, 3415 Bainbridge Avenue, New York, NY 10467 USA; Albert Einstein College of Medicine, 1300 Morris Park Avenue, New York, NY 10461 USA; Division of Hematology-Oncology, Children’s Hospital at Montefiore Medical Center, 3415 Bainbridge Avenue, New York, NY 10467 USA; Department of Radiology, Bronx-Lebanon Hospital, 1650 Grand Concourse, New York, NY 10457 USA; Department of Pathology, Montefiore Medical Center, 111 East 210th Street, New York, NY 10467 USA

**Keywords:** Angiosarcoma, Spleen, Pediatric

## Abstract

Primary splenic angiosarcoma is an extremely rare and aggressive neoplasm of the vasculature. Uniformly, primary splenic angiosarcoma is a fatal disease despite early diagnosis and treatment. Only patients with localized disease amenable to surgical resection achieve long-term, disease-free survival. We present a review of the literature and report a case of a 3-year-old girl with metastatic primary splenic angiosarcoma who was offered aggressive surgical and medical treatment with curative intent despite her advanced presentation.

## Background

Primary splenic angiosarcoma (PSA) is an extremely rare and aggressive neoplasm of the vasculature that occurs in adults and children. Uniformly, PSA is a fatal disease despite early diagnosis and treatment. To date, only patients with localized disease amenable to surgical resection are likely to achieve long-term, disease-free survival. In this review, we summarize the literature and report a case of a 3-year-old girl with metastatic PSA who was offered aggressive surgical and medical treatment with curative intent despite her advanced presentation. Following splenectomy, our patient was given a gemcitabine-docetaxel chemotherapeutic regimen, which has never been described before in children with PSA. This treatment regimen achieved a partial response and was followed by liver metastasectomy. This enabled us to render her free of any gross disease and allowed her to undergo the living-related liver transplantation evaluation as a viable curative option.

## Case presentation

**Figure 1:**
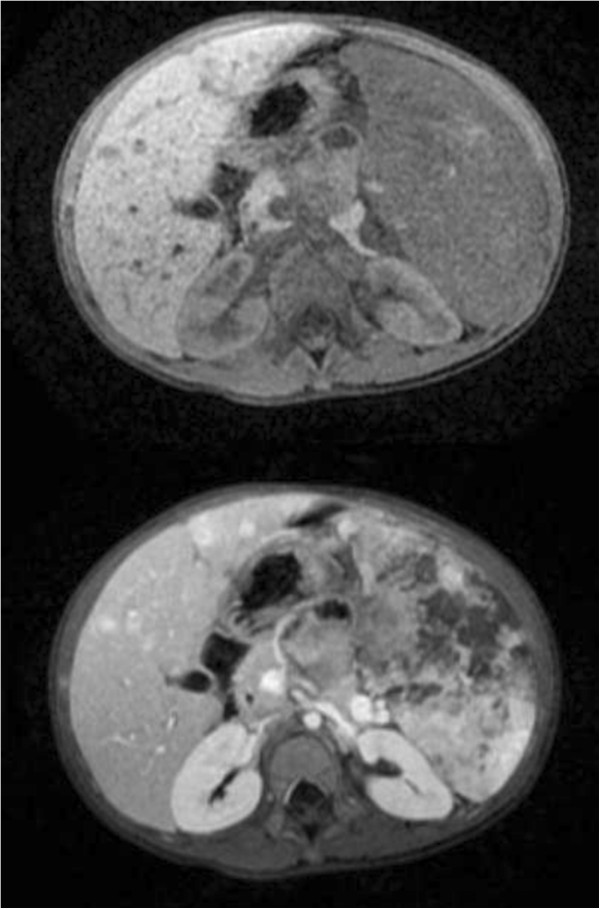
**Axial pre- and post-contrast T1-weighted magnetic resonance images.** The pre-contrast T1-weighted image demonstrates an enlarged spleen with a small area of high signal in the periphery, suggestive of focal hemorrhage. Pre-contrast T1-weighted images also demonstrate three small hypointense hepatic lesions in both lobes of the liver, which show avid enhancement following administration of intravenous contrast. Bottom. Post-contrast T1-weighted images of the spleen demonstrate nearly complete replacement of the spleen by the tumor and by multiple areas of necrosis.

**Figure 2:**
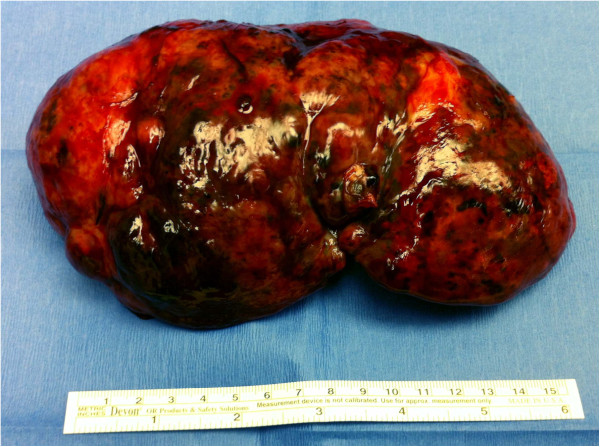
**Gross multinodular spleen specimen.** The tumor is not a well circumscribed mass but rather a diffuse process replacing the entire spleen, therefore a definitive size is difficult to determine. The spleen weighed 540 g and measured 17 × 12 × 45 cm in overall dimensions.

**Figure 3:**
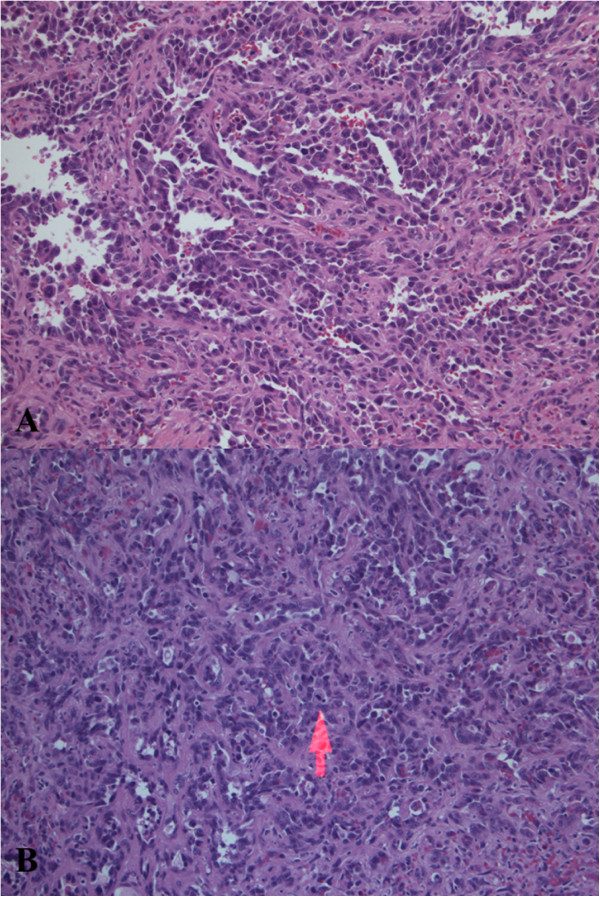
**Histologic examination revealed findings consistent with high-grade angiosarcoma. (A)** Medium power view showing anastomosing vascular channels lined by highly atypical cells (200×). **(B)** Medium power view showing numerous mitoses with one highlighted by the arrow (200×).

**Figure 4:**
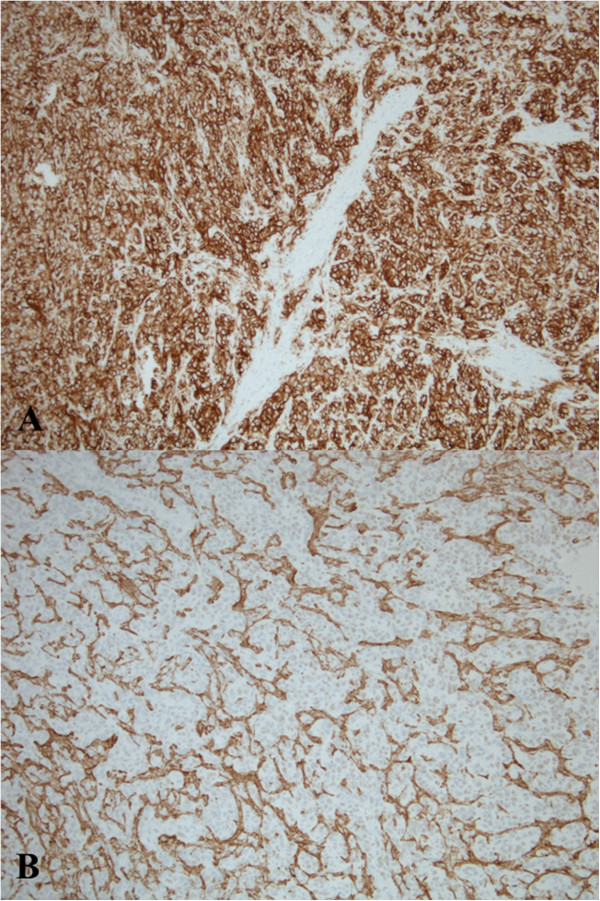
**The diagnosis of primary splenic angiosarcoma was confirmed with additional stains for smooth muscle actin and CD34. (A)** Low power view of strongly positive CD34 stain (100×). **(B)** Low power view of smooth muscle actin stain, which highlights the vascular architecture (100×).

We report on a previously healthy 3-year-old girl who presented with complaints of abdominal pain and distention, and decreased oral intake in the setting of high fevers. On physical examination, she was found to have massive splenomegaly. Her laboratory work-up was significant for anemia and mild thrombocytopenia. An abdominal X-ray showed a markedly enlarged spleen with displacement of bowel and an abdominal ultrasound revealed several hyperechoic nodules in an enlarged spleen. This prompted magnetic resonance imaging (MRI) of the abdomen which showed massive splenomegaly with central hemorrhage and multiple nodular lesions in the liver (Figure 1).Fearing splenic rupture in the setting of hemodynamic decompensation, an emergent splenectomy was performed. Intraoperatively, she was noted to have a massively enlarged multinodular spleen, gross nodular lesions on her liver, a minimal amount of ascites, and no evidence of further organ involvement. Pathologic examination revealed an enlarged, nodular spleen with a central hemorrhagic component (Figure 2). Histologic examination revealed findings consistent with high-grade angiosarcoma (Figure 3) and the diagnosis of PSA was confirmed with additional stains for smooth muscle actin and CD34 (Figure 4). Cytology from the peritoneal fluid was negative for malignant cells.

**Figure 5:**
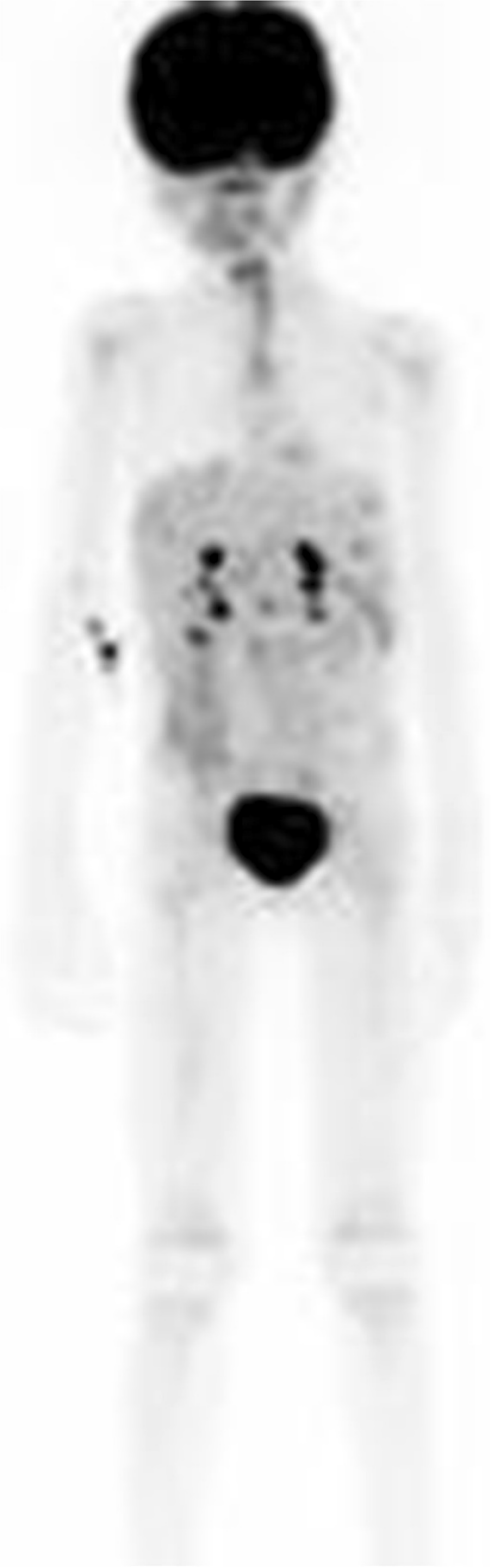
**Positron emission tomography-computed tomography (posterior view) confirms diffuse small areas of uptake in the hepatic parenchyma consistent with metastatic disease to the liver.**

**Figure 6:**
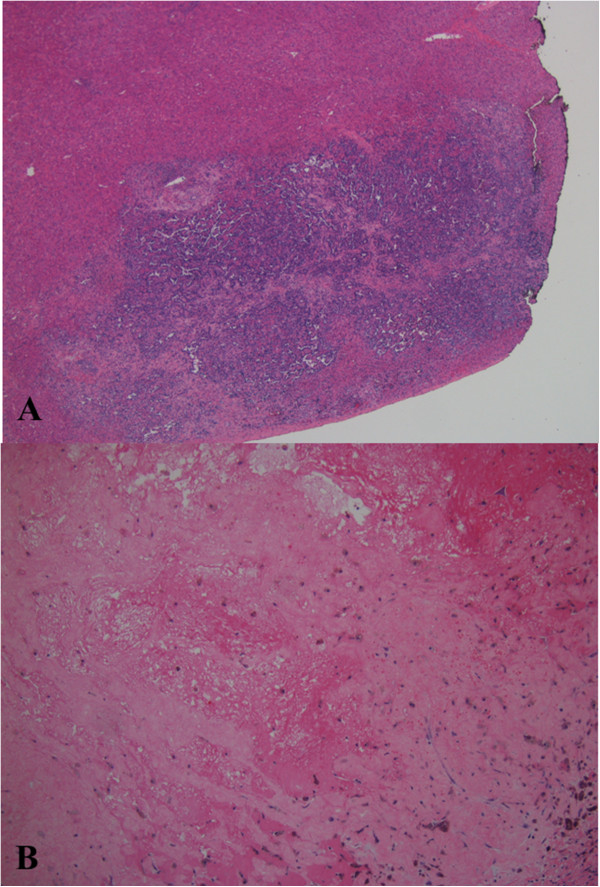
**Histologic examination of wedge liver biopsy. (A)** Low power (2×) view of metastatic liver lesion showing area of necrosis and hemosiderin with a small focus of viable tumor remaining at the periphery of the specimen. **(B)** Low power (4×) view showing area of necrosis and hemosiderin with no viable tumor remaining.

Her post-operative work-up included positron emission tomography-computed tomography (PET/CT), which revealed fluorodeoxyglucose-avid disease exclusive to the liver (Figure 5). Given the diffuse, nodular infiltration of the liver, her disease was deemed unresectable and she was offered chemotherapy. She was treated with a total of eight cycles of gemcitabine and docetaxel as described by Rapkin and colleagues [1]. A repeat MRI after cycle 5 demonstrated a decrease in the size and number of the liver lesions. Five months after her splenectomy, she underwent a wedge liver biopsy to evaluate her metastatic disease. The pathology revealed extensive necrosis with foci of viable tumor cells consistent with a partial response to chemotherapy (Figure 6).

After extensive discussion in our multidisciplinary tumor board, and given her favorable response to chemotherapy, the option of living-related liver transplantation was made available to the family as a means to render the patient disease-free. However, during the transplant evaluation time period, the patient had clinical deterioration and was admitted to the hospital with worsening abdominal pain and distention. She was found to have bilateral pleural effusions and extensive ascites. Liver failure rapidly ensued and, within days of her admission, her family opted for comfort care measures. She died shortly thereafter.

## Discussion

First described by Theodor Langhans in 1879 [2], PSA is among the rarest forms of cancer with a reported incidence between 0.14 and 0.25 per million persons [3, 4]. The median age of diagnosis is 63 years old and, to date, only seven pediatric cases of PSA have been described in the literature (Table 1) [5–11]. A number of environmental agents or predisposing conditions have been associated with the development of PSA in adults, including exposure to ionizing radiation [10], prior chemotherapy for lymphoma [12], or the presence of benign tumors of the spleen - namely hemangiomas or hemangioendotheliomas [5]. However, given its scarce incidence, it is difficult to definitively isolate risk factors.

**Table 1:** **Published cases of pediatric primary splenic angiosarcoma**

Report	Age	Sex	Metastasis	Reported survival
Alt *et al.* [5]	14 months	M	Liver, lung	3 months
den Hoed *et al.* [6]	2 years	F	Liver	30 months
Ferrara *et al.* (1955) [7]	15 years	F	Liver	
Hsu *et al.* [8]	7 years	M	None	16 years
Kren *et al.* [9]	15 years	M	Liver	<1 month
Serrano *et al.* (2014; present study)	3 years	F	Liver	8 months
Sordillo *et al.* (1983) [10]	12 years	F	Liver, bone, bone marrow	<1 month
Wick *et al.* [11]	13 years	M	Liver, bone, serosal surfaces, skin	10 months

F, female; M, male.

To date, only two case series have been published that describe the pathologic behavior of PSA on a combined 68 adult patients [3, 4]. However, clinical and pathologic characteristics can be gleaned from these small studies. Patients commonly present with abdominal pain (83%) and splenomegaly (92%). Constitutional symptoms are not generally a characteristic of PSA, with only 40% of patients exhibiting weight loss, 10% experiencing fevers, and 5% complaining of fatigue. A small minority of patients (13%) will present with splenic rupture which is uniformly considered a grave prognostic factor given the high probability of widely disseminated disease. Hematologic analysis is also inconclusive. Over 90% of patients present with cytopenia, including anemia (49%), thrombocytopenia (25%), or pancytopenia (23%), while a small minority of patients exhibit leukocytosis (18%) [3, 4]. It is unclear whether these features are direct manifestations of the disease or simply indirect correlations of splenomegaly.

Histologically, angiosarcomas are rapidly proliferating, highly infiltrating anaplastic tumors that recur locally, spread widely, and have a propensity for rapid lymph node and systemic dissemination [3, 4, 13]. They arise from splenic vascular endothelium and mesenchymal-derived elongated endothelial cells lining the spleen’s spongy sinusoidal network [13]. Their immediate access to the reticuloendothelial system and their location of origin may in part explain the aggressive nature and behavior of PSA. On gross examination, spleens often weigh >1,000 grams [3]. Macroscopic examination reveals a diffuse replacement of splenic parenchyma by tumor [13]. Microscopically, PSA has a characteristic vascular channel formation with carcomatous stroma [14]. The tumor itself consists of disorganized anastomosing vascular channels lined by large, atypical endothelial cells with significant irregular, hyperchromatic nuclei (Figure 3) [13]. Several reports of PSA have described massive splenic calcifications as a feature of the disease [15, 16]; however, calcifications can also be found in benign processes, making it a nonspecific characteristic for malignancy.

Radiologic features of PSA can range from ambiguous lesions to highly aggressive-appearing tumors in the spleen, often with concomitant metastases at the time of diagnosis [17]. Ultrasound findings of PSA are nonspecific in the majority of cases, but it is usually the first diagnostic test performed in children. The most common ultrasound findings are splenomegaly, splenic heterogeneity, and poorly defined solid and cystic lesions [17]. Areas of hemorrhage and necrosis within the tumor are frequently seen as cystic lesions. Increased Doppler flow may be seen in the more solid echogenic portions of the tumor [18]. On CT, the most common appearance is that of a heterogeneous lesion with areas of low density and necrotic degeneration [17]. Non-contrast CT images may demonstrate areas of high density from recent hemorrhage or calcifications from the hemosiderin deposits [19]. Images performed with intravenous contrast show marked heterogeneous enhancement, necrosis, and hemorrhage. CT evidence of dense ascites is highly suggestive of the spontaneous hemorrhage from splenic rupture. Hypervascular metastatic deposits have been reported in the literature [6, 17]. Most of these are found in the liver, but they have also been described in the lungs, bones and lymphatic organs. The MRI appearance of PSA depends on the extent of the hemorrhage and necrosis in the tumor. Areas of increased and decreased signal intensity may be seen on images obtained with both T1- and T2-weighted pulse sequences depending on the presence of blood products and necrosis [17].

Multiple studies on angiosarcomas have reported the lack of association between histological appearance or grade with survival [20]. It appears that small tumor size (<5 cm) exclusively favors a better prognosis [20]. A multivariate analysis of 55 adult patients with cutaneous and solid-organ angiosarcomas reported mitotic count, tumor size, and mode of treatment as independent favorable prognostic factors [9]. Ultimately, early detection and prompt surgical management is the best approach to prevent splenic rupture, which yields the best chance for survival. Splenectomy prior to splenic rupture has been reported to offer a survival advantage with a mean survival time of 14.4 months versus 4.4 months after rupture [21].

The prognosis for patients with PSA is grim. In the two major case series that examined the behavior of PSA, the overwhelming majority of patients presented with advanced disease [3, 4]. The 6-month overall survival after diagnosis is less than 25%. In the pediatric population, survival has been reported from less than 1 month to 16 years (Table 1). The majority (69 to 100%) of patients with PSA present with metastasis, most commonly to the lungs (78%), liver (63%), bone (31%), and lymph nodes (27%) [3, 4]. These statistics highlight the importance of early surgical intervention in an attempt to achieve long-term disease-free survival [3, 4, 8, 9].

Owing to the rarity of PSA in the pediatric population, the choice of treatment regimen on this particular case was based on the adult angiosarcoma literature. Case reports in adults, while lacking large cohorts of patients, seem to indicate that patients with angiosarcoma respond to gemcitabine and docetaxel, or to other taxanes [11, 22, 23]. The combination regimen of gemcitabine and docetaxel has been shown to be effective in the treatment of various malignancies, including non-small cell lung cancer, breast cancer, and advanced uterine leiomyosarcoma [24–26]. A phase II trial also showed efficacy of this regimen in various metastatic soft tissue sarcomas in adults when compared with gemcitabine alone [27], and, albeit scarce, there is some evidence to suggest that this combination is effective in pediatric soft tissue sarcomas [1, 27].

A retrospective review conducted by Leu and colleagues examined 35 patients with bone and soft tissue sarcomas, including four patients with angiosarcoma [25]. Some patients had previously received chemotherapy, mainly with doxorubicin and/or ifosfamide - a combination often used as first-line therapy in various soft tissue sarcomas - and some received gemcitabine and docetaxel due to the toxicity of the doxorubicin/ifosfamide regimen. Of the angiosarcoma patients, two had a complete response to therapy, one had partial response, and one had stable disease. The patients who had a complete response were chemotherapy-naïve while the others were not.

Additional clinical trials have demonstrated the efficacy of other taxanes at treating angiosarcomas. Penel and colleagues enrolled 30 patients with metastatic or unresectable angiosarcomas in a Phase II clinical trial examining the efficacy and toxicity of weekly paclitaxel [22]. Eight patients had locally advanced unresectable disease; 22 patients had metastatic disease. On this regimen of weekly paclitaxel, the non-progression rate was 74% at 2 months and 42% at 4 months. Overall survival was 56%, 38%, and 21% at 6, 12, and 18 months, respectively. Of interest, after weekly paclitaxel treatment, three patients with local disease showed a complete histological response that made curative-intent surgery feasible [22].

The seven pediatric patients previously described in the literature with the addition of the patient in this case are listed in Table 1 [5–11]. Similar to their adult counterparts, the pediatric patients rarely presented with localized disease, with only one of the eight patients demonstrating disease localized to the spleen. All the rest presented with metastatic disease. Every patient presenting with metastases had liver involvement. Three patients had involvement of other viscera: lung (1), bone (2), bone marrow (1), serosal surfaces (1), and skin (1).

Two of the eight pediatric patients achieved durable remission. The patient with disease limited to the spleen was treated by splenectomy without adjuvant therapy. The patient described by den Hoed and colleagues presented with splenic rupture and liver metastases [6]. She was treated with ifosfamide, vincristine, and actinomycin D for nine cycles with radiographic improvement in the size and number of the liver lesions. After 5 months of observation, two lesions increased in size which prompted wide resection with partial hepatectomy: pathology revealing viable tumor. She received three additional cycles of similar chemotherapy and there remained no evidence of disease at 30 months. Our patient likewise demonstrated radiographic, as well as pathologic, response to chemotherapy. When the liver lesions were biopsied, they revealed primarily necrosis with small pockets of viable tumor. The lesions in the liver subsequently progressed rapidly while awaiting evaluation for liver transplantation. She never developed involvement of her other viscera. Three other patients with metastatic disease were treated with a combination of splenectomy and chemotherapy. All of them succumbed to their disease with a range of a few weeks to 10 months.

## Conclusion

PSA is an extremely rare and aggressive neoplasm of the vasculature that has a very poor prognosis. We present an aggressive multidisciplinary approach in a 3-year-old girl with metastatic PSA who was treated with splenectomy, liver metastasectomy, chemotherapy, and an attempt to liver transplantation. This case report and review of the literature reveals that angiosarcoma in children may be chemo-responsive in some cases. However, this case also illustrates that, in patients with gross disease, surgical extirpation of the primary neoplasm and all metastases is necessary to achieve durable remission. Furthermore, this case underscores the importance of an aggressive multi-disciplinary team approach in these complicated cases aiming for a cure.

## Consent

Written informed consent was obtained from the patient’s family for publication of this case report and any accompanying images. A copy of the written consent is available for review by the Editor-in-Chief of this journal.

## Abbreviations

CT: computed tomography
MRI: magnetic resonance imaging
PSA: primary splenic angiosarcoma.
